# Droplet precautions on-site (DroPS) during the influenza season 2018/2019: a possible alternative to single room isolation for respiratory viral infections

**DOI:** 10.1186/s13756-021-01038-y

**Published:** 2022-01-09

**Authors:** Michèle Birrer, Martin Perrig, Fabienne Hobi, Christina Gfeller, Andrew Atkinson, Martin Egger, Corinne Bartholdi, Drahomir Aujesky, Jonas Marschall, Rami Sommerstein

**Affiliations:** 1 Department of Infectious Diseases, Inselspital, Bern University Hospital, University of Bern, Frohburgstrasse 3, 6002 Luzern, Switzerland; 2 Department of General Internal Medicine, Inselspital, Bern University Hospital, University of Bern, Bern, Switzerland; 3 Hospital Hygiene, Regional Hospital Emmental, Burgdorf, Switzerland; 4 Division of Infectious Diseases, Department of Medicine, Washington University School of Medicine, St. Louis, MO USA; 5 Department Health Sciences and Medicine, Clinic St. Anna, University of Lucerne, Bern, Switzerland

**Keywords:** Isolation, Droplet precautions, Hospital-acquired infection, Respiratory viral infection, Health-care acquired infections, Healthcare epidemiology, Virus, Influenza

## Abstract

**Background:**

The guideline-driven and widely implemented single room isolation strategy for respiratory viral infections (RVI) such as influenza or respiratory syncytial virus (RSV) can lead to a shortage of available hospital beds. We discuss our experience with the introduction of droplet precautions on-site (DroPS) as a possible alternative.

**Methods:**

During the 2018/19 influenza season we introduced DroPS on several wards of a single tertiary care center, while other wards maintained the traditional single room isolation strategy. On a daily basis, we evaluated patients for the development of respiratory symptoms and screened those with a clinical diagnosis of hospital-acquired respiratory viral infection (HARVI) for influenza/RSV by molecular rapid test. If negative, it was followed by a multiplex respiratory virus PCR. We report the concept of DroPS, the feasibility of the strategy and the rate of microbiologically confirmed HARVI with influenza or RSV infection on the DroPS wards compared to wards using the traditional single room isolation strategy.

**Results:**

We evaluated all hospitalised patients *at risk* for a HARVI, 741 (72%) on the DroPS wards and 293 (28%) on the regular wards. The hospital-acquired infection rate with influenza or RSV was 2/741 (0.3%; 1× influenza A, 1× RSV) on the DroPS wards and 2/293 (0.7%; 2× influenza A) on the regular wards.

**Conclusions:**

Droplet precautions on-site (DroPS) may be a simple and potentially resource-saving alternative to the standard single room isolation strategy for respiratory viral infections. Further studies in a larger clinical context are needed to document its safety.

**Supplementary Information:**

The online version contains supplementary material available at 10.1186/s13756-021-01038-y.

## Background

The guideline-driven and widely implemented pathogen-based single room isolation strategy for respiratory viral infections (RVI) such as influenza or respiratory syncytial virus (RSV) [1–3] can lead to a shortage of single rooms in hospitals, especially in times of increased demand (e.g. strong seasonal influenza epidemic, concurrent outbreaks with multidrug-resistant organisms or the ongoing SARS-CoV-2 pandemic). This leads to patient movements that would otherwise not be necessary, is both inconvenient for patients and healthcare workers (HCW), may result in lower quality of care and can cause tangible economic strains for the hospital.

Beyond that, the traditional pathogen-based isolation strategy has notable disadvantages and may even be counter-productive. For example, for patients with RSV infection, some guidelines suggest applying contact isolation precautions [4, 5]. This may cause healthcare workers to omit wearing a mask while close to the patient and increase their risk for nosocomial infections. In rhinovirus and human coronavirus (except MERS-CoV or SARS-CoV-1/-2) infections, isolation precautions are not required in immunocompetent patients, but these viruses can lead to nosocomial transmissions as well [6, 7]. In addition, any pathogen-based isolation strategy will depend on costly diagnostic tools.

Hospital-acquired respiratory viral infections (HARVI) have been shown to be responsible for ~ 12% of severe hospital acquired pneumonias [6] and may affect ~ 16,000 adults in US acute care hospitals per year [8]. Of note, HARVI other than influenza and RSV are quite common and probably have an underestimated impact on patient morbidity and mortality [6, 7, 9].

Novel strategies that focus on coping with the shortage of single rooms and reducing patient risk from all respiratory viruses are needed. A pragmatic preventive approach to respiratory viral diseases was introduced a few years ago in several Swiss regional hospitals. Regardless of the pathogen, patients with symptoms of a respiratory infection were placed on droplet precautions at the patient bed site. According to preliminary data, the rate of hospital-acquired influenza infections was comparable to that of other Swiss hospitals that followed the standard single room isolation approach [10].

Based on this experience, in the 2017/2018 influenza season, the concept of droplet precautions on-site (DroPS) was introduced in four hospitals affiliated with Bern University Hospital, where there had been no comprehensive HARVI prevention strategy previously. In a survey, healthcare workers rated the DroPS strategy as good in terms of acceptability, safety and comprehensibility [11].

After our institution was affected by a large VRE outbreak [12] and in light of a foreseeable shortage of single rooms for the influenza season 2018/2019, the hospital infection prevention committee decided to implement the DroPS strategy on certain wards of the Department of General Internal Medicine of Bern University Hospital and test it against the standard strategy.

The aim of this pilot study was to introduce DroPS, assess its feasibility and compare the rate of HARVI between the DroPS strategy and the traditional single room isolation strategy during the influenza season 2018/19.

## Methods

### Setting

This single center, pragmatic, pilot study was conducted at the Department of General Internal Medicine, Bern University Hospital, Switzerland, a 950-bed tertiary care center. The department runs a total of 97 beds on six wards. Patients were enrolled from 17.01.2019 to 16.04.2019, the time period where influenza cases exceeded the epidemic threshold (68/100′000 patients/week) on a national level [13].

### Introduction of DroPS

DroPS was introduced on four wards (totaling 73 beds, including 5 single rooms, 24 twin rooms, 2 four-bed and 2 six-bed rooms). Two wards (totaling 24 beds, including 4 single rooms, 2 twin rooms, 4 four-bed rooms) were defined as “regular” wards, where the pathogen-based single room isolation strategy was maintained. Based on the total number of beds, this corresponds to a ratio of 3:1.

The allocation of patients to the wards was done by the hospital bed managers, driven by bed availability.

#### DroPS strategy

**Fig. 1:**
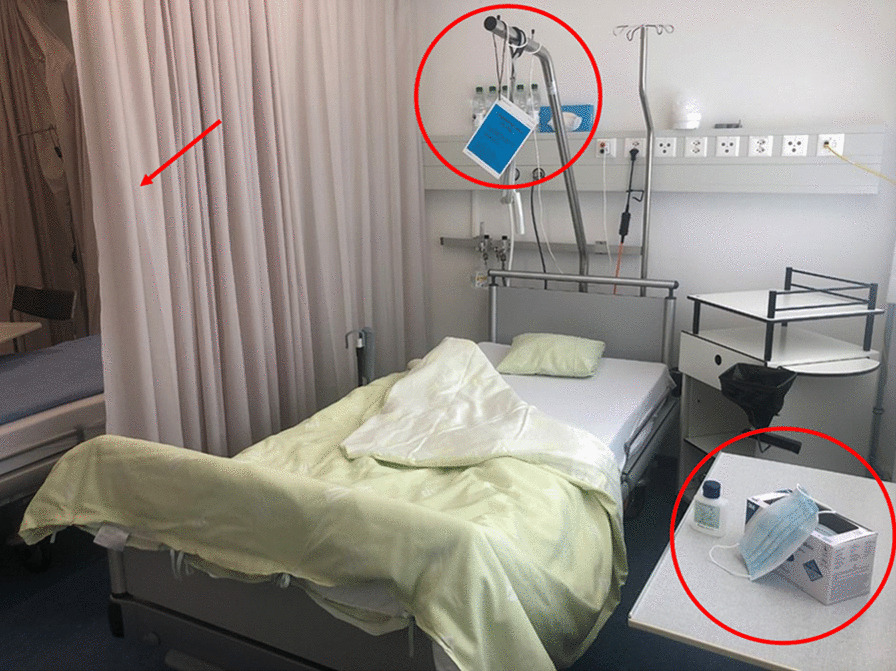
Example of the typical features of a DroPS patient bed site: curtain drawn next to patient bed; signage of the patient bed; distribution of surgical masks and bedside disinfectant to the patient

DroPS was introduced at the patient bed site based on clinical criteria. DroPS could be started by any HCW (including those in training) if a patient presented new respiratory symptoms (cough, rhinitis, sore throat) and/or was diagnosed with pneumonia, exacerbated COPD or acute bronchitis. The respiratory pathogen – if known—had no influence on the approach taken. DroPS included the following: signage of the patient bed; curtain drawn next to patient bed; distribution of surgical masks and bedside disinfectant to the patient including information about its use when leaving the bed site (Fig. 1); HCW wearing a surgical mask if patient contact < 1.5 m of distance; and enforcement of standard hygiene precautions. Immunocompromised, non-cooperative and non-compliant patients were excluded from DroPS, as well as patients with suspected bacterial meningitis, pertussis, rubella or mumps. A detailed description of all DroPS measures is provided in Additional file 1: Table S1. The introduction of DroPS was preceded by extensive training and instruction of the nursing teams and physicians.

#### Standard pathogen-based single room strategy

On the regular wards, based on the detected viral pathogen, respiratory precautions were either continued as droplet or contact isolation in a single room or transformed into standard hygiene precautions, according to the institutional policy (Additional file 1: Table S2).

The isolation precautions on both the DroPS and regular wards were stopped as soon as the patient was no longer symptomatic or a reduction of symptoms to the individual’s baseline occurred.

### Data sampling

For each hospitalisation, we collected admission date, patient identifier, case identifier, date of birth, gender, ward, room number and respiratory isolation status once daily (at 12:00 p.m.). Swab results and hospitalisation duration was retrieved from the digital patient management system.

There was continuous risk assessment of DroPS through monitoring and daily analysis of every possible HARVI during the assessment period.

### Definitions/outcomes

#### Inclusions and exclusions

For the descriptive outcomes we included the patient population considered *at risk* for HARVI, comprising all hospitalisations with no respiratory isolation precautions during the first two days after admission. Excluded were hospitalisations with a transfer from DroPS to regular wards and vice versa (i.e. cross-over hospitalisations).

#### Diagnostics

**Fig. 2:**
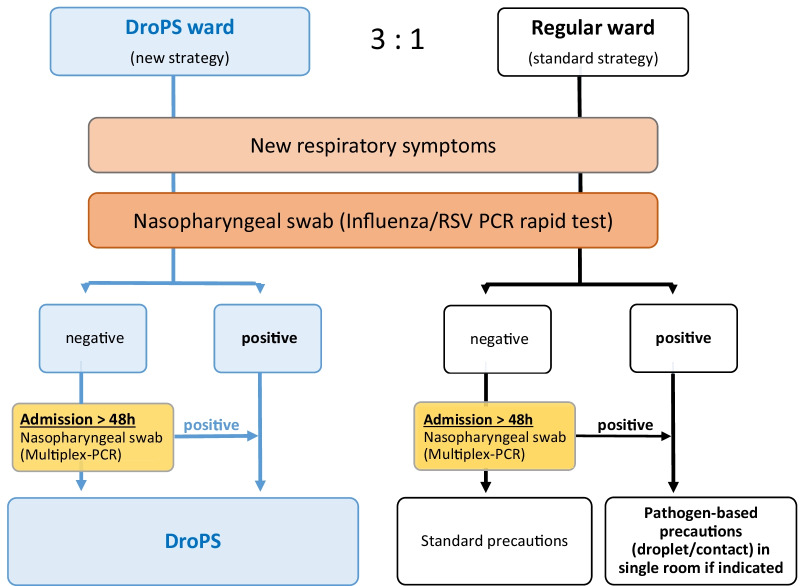
Algorithm for diagnostics and respiratory precaution measures on DroPS versus regular wards. DroPS = Droplet precautions on-site; influenza/RSV PCR = molecular rapid test for influenza and RSV (respiratory syncytial virus); multiplex-PCR = multiplex respiratory virus PCR testing for adenovirus, rhinovirus, coronavirus (not MERS or SARS-CoV), human metapneumovirus and human parainfluenza virus

On a daily basis, patients on all wards were screened for the onset of new respiratory symptoms. If present, a nasopharyngeal swab and influenza/RSV molecular rapid test was performed (Cobas® LIAT®, Roche, Switzerland). If negative, and if the onset of symptoms was hospital-acquired, this test was followed by a nasopharyngeal swab for multiplex respiratory virus PCR, directed at adenovirus, rhinovirus, coronavirus (not MERS or SARS-CoV), human metapneumovirus and human parainfluenza virus (from ARGENE® Respiratory menu, Biomérieux, France) (summarized in Fig. 2).

#### Definition of hospital-acquired viral infection (HARVI)

We evaluated patients for HARVI who developed acute respiratory symptoms after two days (i.e. ≥ day 3) after admission to one of the DroPS or regular wards (see Additional file 1: Table S3), in line with the definition of nosocomial infections, which is characterised as infection occurring 48 h or later after hospital admission [14].

#### Primary and secondary exploratory outcome

The primary composite outcome was the rate of hospitalisations with microbiologically confirmed HARVI with influenza or RSV in the patient population *at risk.*

Secondary outcomes were the rate of hospitalisations with microbiologically confirmed HARVI due to any respiratory viruses; the rate of hospitalisations with clinical HARVI and the rate of hospitalisations with clinical HARVI, either microbiologically confirmed or with missing test, in the patient population *at risk*.

Adjustments for the following variables were planned: patient characteristics; isolation strategy; data from the previous four hospitalisation days of the patient: single vs. multi-bed room, isolation rate / ward, isolation rate / room, isolation type of the patient and influenza vaccination rate of HCW.

### Sample size

Based on the previous year's hospitalisation data, the rate of HARVI with influenza and RSV during the influenza period was estimated to be 0.7% (data not shown). Assuming similar patient numbers to the previous year, we estimated to include approximately 750 patient hospitalisations in the DroPS and 300 in the regular group. With this number of expected patients, a statistical significance level of 5%, and 80% power, we estimated that we would be able to detect a non-inferiority margin of approximately 1.3%. This means we considered a HARVI rate up to (0.7 + 1.3 =) 2% (i.e. the upper 95% confidence interval below 1.3%) in the DroPS group to be similar to the rate of 0.7% in the regular group (calculated using function “TwoSampleProportion.NIS” in package “TrialSize” in R [15]).

### Statistics

Categorial variables are presented as number (percentages) and for continuous variables as median (interquartile range; IQR). Differences between DroPS and regular groups were investigated using the chi-square test (or variants thereof) for categorical variables and the non-parametric Wilcoxon rank sum test for continuous variables.

The primary outcomes were the unadjusted rate of confirmed HARVI with influenza or RSV on the DroPS and regular wards in the patient population *at risk*. We further described the risk difference between the DroPS and regular wards for the outcomes of confirmed HARVI with influenza or RSV. Analysis of further outcomes followed the same approach.

A level of 5% was considered statistically significant throughout. All analyses were performed using R version 3.6.1 [16].

## Results

**Fig. 3:**
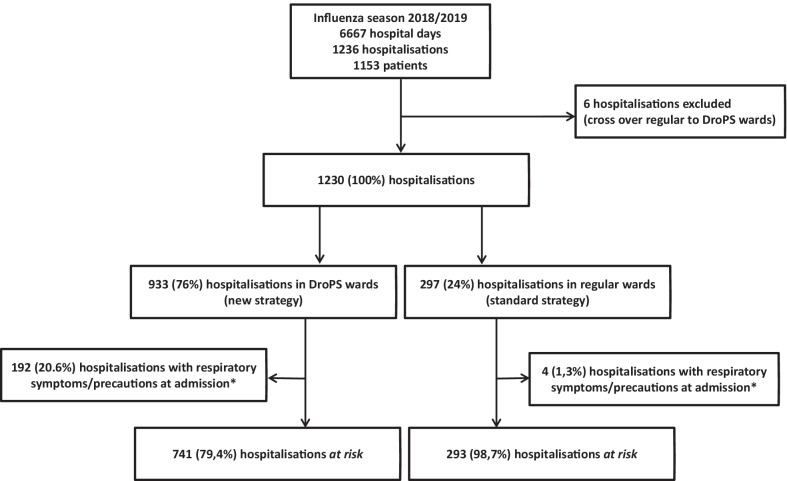
Flow diagram of all hospitalisations on DroPS and regular wards during the influenza season (17.01–16.04.2019). DroPS = Droplet precautions on-site. * respiratory symptoms/precautions at admission means during the first two days of hospitalisation on DroPS or regular ward, i.e. day 0, 1 or 2

There were a total of 1230 hospitalisations in the Department of General Internal Medicine from 17.01.2019–16.04.2019, 933 (76%) on the DroPS wards, 297 (24%) on the regular wards (Fig. 3).

### Exclusions

On admission, 192/933 (20.7%) patients on the DroPS wards were placed on precautions due to respiratory symptoms, versus 4/297 (1.3%) patients on the regular wards. From all patients with precautions upon admission (i.e. during the first two days of hospitalisation on the wards), a viral infection with influenza or RSV was detected in 102/192 (53%) patients on the DroPS wards, versus 4/4 (100%) on the regular wards (Additional file 1: Table S4). This patient population was excluded from the outcome analysis.

Six cross-over hospitalisations with a transfer from the regular to the DroPS wards during their hospitalisation were also excluded.

### Outcomes

We included 1034 hospitalisations *at risk*, 741/933 (79.4%) on the DroPS wards, 293/297 (98.7%) on the regular wards (Fig. 3).

Baseline and hospitalisation characteristics are summarized in Table 1. The median age was similar on both wards (DroPS 71 years (IQR 57–82) vs. regular 72 years (IQR 56–80); *p* = 0.61). The proportion of females was lower on the DroPS wards (DroPS 42.9% vs regular 50.8%; p = 0.025). The total of hospital days spent in multi-bed rooms was significantly higher on the DroPS wards than on the regular wards (DroPS 3635 (92%) vs regular 1366 (82%); *p* < 0.001).

**Table 1:** Baseline and hospitalisation characteristics for included patients (i.e. hospitalisations *at risk* for hospital-acquired respiratory viral infection)

Characteristic	DroPS wards	Regular wards	P value
Patients (hospitalisations), no	741	293	
Age in years, median [IQR]	71 [57, 82]	72 [56, 80]	0.617
Female sex, no. (%)	318 (42.9)	149 (50.9)	0.025
**Hospital days**			
Total no	3944	1659	
Per hospitalisation,median [IQR]	4 [2, 7]	5 [2, 7]	0.063
In multi-bed rooms, no. (%)	3635 (92%)	1366 (82%)	< 0.001

[IQR] = interquartile range; no. = number; % = percent

For the primary outcome, HARVI with influenza or RSV, there was no statistically significant difference between the two groups: On the DroPS wards, the microbiologically confirmed rate of influenza/RSV infections was 2/741 (0.3%; 1× influenza A, 1× RSV), compared to 2/293 (0.7%; 2× influenza A) on the regular wards (risk difference -0.4%, 95% CI − 1.6 to 0.8; *p* = 0.68) (Table 2). The confidence interval of the HARVI rate in the DroPS group did not cross the pre-specified margin of 1.3%, so formally non-inferiority was confirmed.

**Table 2:** Hospital-acquired respiratory viral infections (HARVI) in patients *at risk*

Outcome, no (%)	DroPS wards (n = 741)	Regular wards (n = 293)	Risk difference in % (95% CI)	*P* value
**Primary outcome**				
Influenza/RSV positive, no. (%)	2 (0.3)	2 (0.7)	−0.4 (− 1.6 to 0.8)	0.684
**Secondary outcomes**				
Any respiratory virus positive, no. (%)	5 (0.7)	2 (0.7)	0.0 (− 1.1 to 1.1)	0.9
Clinical diagnosis, no. (%)	17 (2.3)	2 (0.7)	1.6 (− 0.05 to 3.2)	0.138
Influenza/RSV positive – or no Influenza/RSV test performed, no. (%)	4 (0.5)	2 (0.7)	−0.2 (− 1.3 to 1.1)	0.9
Any respiratory virus positive – or no complete viral panel test performed, no. (%)	9 (1.2)	2 (0.7)	0.5 (− 0.9 to 2.0)	0.678

No. = number; (%) = percent; RSV = respiratory syncytial virus; DroPS (droplet precautions on-site); regular wards: pathogen-based single room strategy. Positive risk difference indicates regular is better than DroPS

All microbiologically confirmed HARVI with influenza occurred at day 4 or 5 of hospitalisation; the HARVI with RSV at day 11. A detailed description of the presumed transmission mode for each HARVI is found in the Additional file 1: Table S5.

**Fig. 4:**
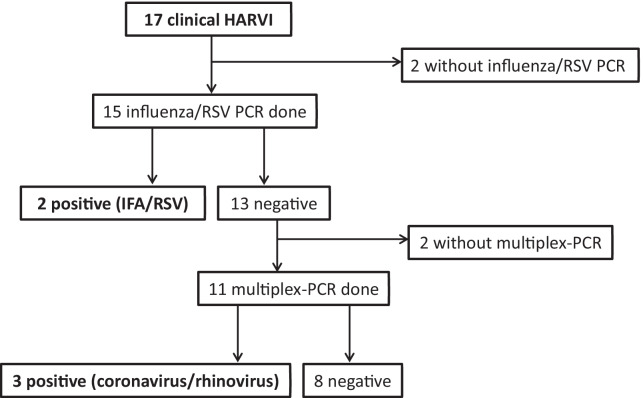
Overview: microbiological testing for clinically diagnosed hospital-acquired respiratory viral infections (HARVI) in the DroPS wards. Influenza/RSV PCR = molecular rapid test for influenza and RSV (respiratory syncytial virus); multiplex-PCR = molecular test for adenovirus, rhinovirus, coronavirus (not MERS or SARS-CoV), human metapneumovirus and human parainfluenza virus

For the secondary outcome, HARVI with any respiratory virus, three additional human coronavirus infections (in one case coinfection with rhinovirus) were identified on the DroPS wards, resulting in 5/741 (0.7%) versus 2/293 (0.7%) on the regular wards (risk difference 0.0%, 95% CI − 1.1 to 1.1; *p* = 0.9). There were 17/741 (2.3%) clinically diagnosed HARVI on the DroPS wards compared to 2/293 (0.7%) on the regular wards (risk difference 1.6%, 95% CI − 0.05 to 3.2; *p* = 0.13). On the DroPS wards, two patients with clinical HARVI did not undergo any microbiological testing, while in two additional cases only detection tests for influenza/RSV were performed, but not for other respiratory viruses. An analysis that regarded patients with clinical HARVI diagnosis without complete diagnostic testing as worst case (i.e. having a microbiologically proven HARVI) did not reveal a significant difference between the DroPS and regular wards either (Table 2, Fig. 4).

The primary and secondary outcome events were very rare, therefore it was not possible to adjust our results for independent variables (patient characteristics; isolation strategy; data from the previous four hospitalisation days of the patient: single vs. multi-bed room, isolation rate/ward, isolation rate/room, isolation type of the patient and influenza vaccination rate of HCW).

## Discussion

We evaluated the introduction of droplet precautions on-site (DroPS) versus the standard pathogen-based single room isolation strategy on the rate of hospital-acquired respiratory viral infections (HARVI) during one influenza season. We did neither identify a higher rate of microbiologically confirmed nor of clinically diagnosed HARVI on the DroPS wards compared to the regular wards. Data was collected on a daily basis by a dedicated team and included a detailed analysis of every possible HARVI. DroPS could represent a novel and straightforward hospital-wide strategy for dealing with respiratory viral infections in hospitalised patients. Based on the results of this pilot study we think that DroPS is a potentially promising alternative to the standard single room isolation strategy, although yet to be validated in a larger clinical trial.

### Impact of DroPS

#### Healthcare system

DroPS is a symptom-based strategy and therefore potentially resource-saving; compared to a pathogen-based isolation strategy, there is - from an infection control point of view - no a priori need for diagnostics, thus allowing for lower costs. It constitutes a flexible operating system where measures can be started by any HCW without the need for advice from the infection prevention team. In addition, time-consuming patient transfers (to single rooms) and related cleaning measures are likely reducible. The implementation of DroPS could be a solution for infection prevention in settings with limited resources and may reduce the pressure on single rooms, especially if they are needed for other reasons (i.e. outbreaks). As such, DroPS may be interesting from an economical point of view.

#### Safety

One HARVI with RSV on a DroPS ward deserves special attention. It seemed likely that a fellow patient, hospitalised with an RSV infection in the same room, was the source of transmission. This could question the safety of DroPS, as the strategy only requires contact measures according to standard hygiene precautions. Therefore, one could argue that the risk for hospital-acquired RSV transmissions could increase due to omission of contact precautions. We nevertheless believe that contact barriers are well ensured with DroPS, due to the reinforcement of standard precautions (i.e. hand hygiene, wearing gloves and gown if contact with body fluids/substances, see Additional file 1: Table S2), and that a symptom-based approach for respiratory precautions may provide additional protection against those respiratory viruses that otherwise would not result in specific precaution measures (e.g. rhinovirus [6]).

The DroPS strategy is based on symptoms and therefore does not provide protection against HARVI in case viral transmission occurs from an asymptomatic source. For influenza, some data indicate that the transmission in the presymptomatic stage is of little relevance in the hospital setting [17].

We did not evaluate, how often patients on the DroPS wards had to be transferred to a single room after initiation of DroPS or were initially assigned to the regular wards because of severe immunosuppression, anticipated poor adherence to and/or contraindications for DroPS. Our data does not suggest that a hypothetical poor adherence to measures by patients on DroPS wards led to a worse outcome (in which case we would have expected to see more HARVI cases on DroPS wards).

#### HCW

The significant difference in the rate of respiratory precautions started upon admission between the DroPS and the regular wards points to a preferred admission of patients with suspected RVI to the DroPS wards. This may indicate that DroPS is a convenient concept that facilitates inpatient bed management. Furthermore, as already reported previously [11], a high level of appreciation of DroPS in terms of comprehensibility, acceptance and safety was seen in HCW (data not shown).

There was a trend towards more clinical HARVI (i.e. new respiratory symptoms diagnosed more than two days after admission without microbiological evidence of infection) on the DroPS wards. The implementation of DroPS may have lowered the threshold to start precaution measures, given that the sole trigger was the presence of respiratory symptoms, possibly leading to DroPS in patients with symptoms due to non-infectious etiologies (i.e. cough because of cardiac insufficiency, post-interventional spasms after bronchoscopy). This may indicate that DroPS can lower the threshold for respiratory precautions and therefore result in a higher number of inpatients placed on respiratory precautions compared to the standard strategy.

#### Patients

The following concerns were reported as relevant by the treating HCW: There was an increased need for information and education about DroPS to the non-isolated fellow patients in the same room and their relatives. Of note, some patients with DroPS also did not appreciate being shielded with curtains from their fellow patients.

### Power calculation/non-inferiority

Both the rate of HARVI and the number of hospitalisations were approximately the same in the study period compared to those in the previous years, and therefore the study was adequately powered to detect the pre-specified non-inferiority margin of 1.3%.

From a clinical point of view, it can be argued that a rate of 0.7% on the regular wards and 2% on the DroPS wards should not be considered similar. Translated into clinics, this would mean that a HARVI rate in the DroPS group would only be considered unacceptably high if it exceeded 2%. Or, in other terms, that an approximate doubling (increase of 1.3%) of the HARVI rate would still be acceptable.

Whether these potential losses in patient safety could be outweighed by the advantages of DroPS (preservation of hospital capacity, conservation of resources of the healthcare workers, and a simple and pragmatic respiratory precautions strategy) is a question of preferences set by the hospital leadership and its prioritization is a matter of discretion.

Retrospectively, it can also be debated whether a non-inferiority design is adequate to address the question of this pilot study.

### Limitations

#### Data

Several limitations and biases affect the internal validity of our data. First, it was a pragmatic, single department pilot study without cluster randomization. Second, an allocation bias is likely, as patients admitted with probable respiratory viral infections (RVI) were more frequently directed to the DroPS wards. Third, the primary and secondary outcome events were very rare, and thus adjusting for possible confounding variables was not possible. Fourth, for the patient population *at risk* in the DroPS group, we did not analyse how many were actually exposed to symptomatic fellow patients in the same room. Fifth, the use of a qualitative test method (here: PCR without measurement of cycle thresholds) lacks information about viral load as an indirect marker for infectivity. Therefore, transmission risk from admitted patients with influenza/RSV infections remains ill-defined. Sixth, we did not assess HCW absenteeism or infections with influenza/RSV nor did we monitor RVIs in visitors, which could be another confounder. Seventh, a potential exclusion bias lies in the focus on the study population *at risk* for HARVI. On the DroPS wards ~ 20% of all hospitalisations had respiratory precautions on admission and were excluded from the outcome analysis. We cannot rule out the possibility of additional HARVIs occurring in this population, because patients with pre-existing respiratory symptoms and already on precautions were not screened for “new” respiratory infections. We believe that including these patients would have caused substantial methodological issues while not changing the results. Lastly, and of importance, we cannot exclude missed HARVIs in both groups because we did not exclude patients with a short hospital stay (i.e. shorter than the expected incubation period for influenza and RSV) and did no follow-up of the patients after discharge.

#### Concept

First, DroPS as a concept does not cover transmission through smaller air particles (i.e. aerosols) which must be considered under certain circumstances (e.g. oxygen high flow therapy, or infections with, for example, SARS-CoV-2 [18]). During the COVID-19 pandemic, DroPS continued to be used in our institution but only for patients for whom SARS-CoV-2 infection was ruled out. Second, even if it may not be needed from an infection prevention point of view, clinicians might want to continue diagnostic testing for viral pathogens other than SARS-CoV-2 because of therapeutic implications, especially in high-risk patients.

## Conclusions

This pragmatic pilot study demonstrated the feasibility of introducing the droplet precautions on-site strategy to a tertiary referral center. It suggests that DroPS is a promising—yet formally unproven—alternative strategy to standard single room isolation in hospitalised patients with respiratory viral infections. Of note, it remains to be seen if DroPS also works in the context of a pandemic respiratory virus, such as SARS-CoV-2 (19).

As next step, a multi-center, cluster-randomized study with complete post-discharge follow-up should be pursued to determine the clinical utility and safety of DroPS as a novel approach to HARVI prevention.

## Supplementary Information

**Additional file 1**. DroPS for the prevention of respiratory viral infection.

## Data Availability

The dataset generated and analysed during the current pilot are not publicly available due to reasons of sensitivity (e.g. human data) but are available from the corresponding author on reasonable request.

## Abbreviations

DroPS: Droplet precautions on-site
COPD: Chronic obstructive pulmonary disease
COVID-19: Coronavirus disease 2019
HARVI: Hospital-acquired respiratory viral infection
HCW: Healthcare worker
MERS-CoV: Middle east respiratory syndrome coronavirus
PCR: Polymerase chain reaction
RSV: Respiratory syncytial virus
RVI: Respiratory viral infection
SARS-CoV/-2: Severe acute respiratory syndrome coronavirus / type 2
VRE: Vancomyine-resistant enterococci
