# Supplementary material for: Droplet precautions on-site (DroPS) during the influenza season 2018/2019: a possible alternative to single room isolation for respiratory viral infections
Source: Antimicrob Resist Infect Control. 2022 Jan 9;11:2. doi: 10.1186/s13756-021-01038-y (PMC8743058; doi:10.1186/s13756-021-01038-y)
Supplement: Supplementary file 1 — Additional file 1. DroPS for the prevention of respiratory viral infection. [file 13756_2021_1038_MOESM1_ESM.doc]

**SUPPLEMENTARY**

**Supplementary Table S1**

**Description of the concept Droplet Precautions on-site (DroPS)**

DroPS includes the following: signage of the patient bed regarding isolation precautions; privacy curtains; information of the patient on the isolation; distribution of surgical masks to the patient, to be worn when leaving the bed; HCW wearing a surgical mask if patient contact <1.5m of distance; enforcement of standard hygiene precautions (for an example of a patient bed site, see Figure 1 in the main manuscript).

A maximum of two patients with DroPS/room were allowed. Severely immunocompromised, non-cooperative and non-compliant patients were excluded from DroPS.

The respiratory isolation status of each patient was evaluated on a daily basis at noon by a dedicated team, consisting of infection control nurses and physicians, who obtained the information during ward rounds from the ward staff (HCW) and the documentation in the electronic patient charts.

| Isolation: | - Patients with acute respiratory symptoms (cold, cough, rhinitis, bronchitis, sore throat) or diagnosed with pneumonia or exacerbated copd are isolated at the patient bed site  - Droplet precautions on-site can be started by the nursing staff without consultation of a physician |
| --- | --- |
| Diagnostics: | - No diagnostic procedure is necessary to initiate/terminate isolation precautions |
| Room: | - No single room is required, precautions are established on-site  - Maximum of two patients with DroPS / room allowed |
| Flagging: | - Patient beds are marked with a sign "droplet precautions on-site " |
| Standard precautions: | - Hand hygiene, gloves and gowns with every contact with body fluids/substances  - Surgical mask: Staff and visitors always wear a surgical mask when in direct contact (<1.5 m) with the patient |
| Patients with respiratory symptoms: | - Use paper tissues when sneezing, blowing the nose and coughing, dispose of them immediately after use and then wash or disinfect hands thoroughly  - Wear a surgical mask when leaving the patient's site (waiting areas, examinations) |
| Shared rooms: | - Immobilized patients: Pulling the curtain or placing a screen at the head end of the bed to prevent droplet contamination of the patient environment.  - Partially mobile patients: Place patients in a way that coughing or sneezing cannot contaminate the patient environment; instruct fellow patients to keep a distance (≥1.5 m from head to head) and not to have physical contact; do not seat patients at the room table together.  - Mobile, cooperative patients: Instruction in the leaflet “droplet precautions on-site”: keep a distance (≥1.5 m), no physical contact with fellow patients, no communal meal at the table, application of respiratory etiquette (cough in tissue, dispose of handkerchief, hand disinfection), wear surgical mask when leaving the bed site.  - Fellow patients: No immunosuppressed patients (neutropenia <0.5 G/L, active leukemia or lymphoma, HIV with CD4< 200 c/μl, splenectomized patients, <4 weeks post-transplantation, cytotoxic chemotherapy, high-dose steroids (>2 weeks prednisone equivalent >20mg/d) in the same room as patients with droplet precautions on-site in place. |
| Information: | - Inform transport service, examination units, surgical unit |
| Duration: | - Isolate until respiratory symptoms have subsided or have returned to baseline levels (e.g., in the case of chronic bronchitis).  - Discontinuation of isolation precautions may be ordered by the supervising care team without consultation of the infection control team. |
| Cleaning staff: | - Cleaning staff must be informed about the isolation measure  - Cleaning at discharge: Standard procedure |
| Visitors with respiratory symptoms: | - Are asked not to visit patients and are not allowed to visit immunosuppressed patients. They wear a surgical mask in waiting areas.  - The following precautions are taken in the event of an unavoidable patient visit: Keep your distance from the patient when sneezing, blowing and coughing, use paper handkerchiefs, dispose of them immediately and then wash/disinfect your hands thoroughly. When approaching the patient <1.5 m wear a surgical mask. If physical contact is essential: Hand disinfection before and after patient contact.  - Children with a cold or respiratory infections under 10 years of age are not allowed to visit the hospital, since they may not fully comply with the infection prevention recommendations. |
| Contraindication: | - For severely immunosuppressed, non-cooperative, confused and/or demented patients, droplet isolation in a single room is mandatory.  - If bacterial meningitis, pertussis, rubella or mumps are suspected, droplet isolation in a single room is mandatory. |

**Supplementary Table S2**

**Isolation measures on the regular wards according to institutional policy**

Patients (admitted) with respiratory symptoms receive droplet isolation and a nasopharyngeal swab for influenza A/B and RSV (molecular rapid test) is performed. Diagnostic testing is recommended in our hospital policy for patients admitted with suspected respiratory viral infections according to an internal “case definition” for influenza-like illness (see below).

Criteria for “case definition” of influenza-like illness met if: minimum of 1 major and 1 minor present.

| **Major:** |
| --- |
| - Acute symptoms (< 7 days) |
| - Fever |
|  |
| **Minor:** |
| - Cough |
| - Dyspnea |
| - Sore throat |

At the discretion of the clinician, diagnostics are extended to other viruses (PCR for rhinovirus, adenovirus, coronavirus, parainfluenza virus, human metapneumovirus). Further isolation measures (droplet versus contact) depend on the diagnosed virus and imply hospitalisation in a single room.

| **Viral pathogen** | **Isolation measure** |
| --- | --- |
| Influenza | Droplet isolation |
| RSV | Contact isolation |
| Human Metapneumovirus | Contact isolation |
| Human parainfluenza virus | Contact isolation |
| Adenovirus | Droplet isolation |
| Coronavirus | Standard precautions*/** |
| Rhinovirus | Standard precautions*/** |

* contact isolation precautions if immunosuppressed

** Standard precautions imply: hand hygiene, gloves and gowns with every contact with body fluids/substances, surgical mask when in direct contact (<1.5 m distance).

**Supplementary Table S3**

**Illustration of the assessment and definition of a possible hospital-acquired respiratory viral infection (HARVI)**

The day of hospital admission is always day 0 (time range 00:01 - 23:59).

The presence/absence of respiratory precautions for each hospitalised patient is recorded daily at 12:00 pm. The first day of respiratory precautions is defined as the first day of hospitalisation where the patient has respiratory precautions at noon (12:00 pm), although the real starting time of respiratory precautions may be earlier (time range 0 to -23h 59min).

If the difference between hospital (or ward) admission and the first day of respiratory precautions is more than two days (this means after days 0, 1 and 2), the patient is considered as having a possible HARVI. In other words, the earliest timepoint during a hospitalisation where the definition of a possible HARVI applies is day 3.

The following four examples (A – D) illustrate the variety of the time spectrum that lies between timepoint of admission to the hospital (or ward) and the real start of respiratory precautions (min. 36h, max. 83h) in a patient being recorded as having a possible HARVI.

|  |  | **day 0** | **day 1** | **day 2** | **day 3** | **Time difference between admission and start of respiratory precautions** |
| --- | --- | --- | --- | --- | --- | --- |
| A* | Hospital/ward admission | 23:59 pm |  |  |  | 36h 2min |
|  | Start respiratory precautions |  |  | 12:01 pm |  |
| B** | Hospital/ward admission | 23:59 pm |  |  |  | 50h 1min |
|  | Start respiratory precautions |  |  |  | 12:00 pm |
| C*** | Hospital/ward admission | 00:01 am |  |  |  | 60h |
|  | Start respiratory precautions |  |  | 12:01 pm |  |
| D**** | Hospital/ward admission | 00:01 am |  |  |  | 83h 59min |
|  | Start respiratory precautions |  |  |  | 12:00 pm |

*A: Hospital/ward admission late on day 0, shortly before midnight (23:59); start of respiratory precautions on day 2 just after the recording time at noon (12:01), which means that this patient will only be recorded as having respiratory precautions the following day (= day 3).

**B: Hospital admission late on day 0, shortly before midnight (23:59); start of respiratory precautions on day 3 at the timepoint of recording (12:00).

***C: Hospital admission as early as possible on day 0, shortly after midnight (00:01); start of respiratory precautions on day 2 just after the recording time at noon (12:01), which means that this patient will only be recorded as having respiratory precautions the following day (= day 3).

****D: Hospital admission as early as possible on day 0, shortly after midnight (00:01); start of respiratory precautions on day 3 at the timepoint of recording (12:00).

**Supplementary Table S4**

**Baseline and hospitalisation characteristics for all hospitalisations**

| **Characteristic** | **DroPS wards** | **Regular wards** | **P value** |
| --- | --- | --- | --- |
| Patients (hospitalizations), no. | 933 | 297 |  |
| Age in years, median [IQR] | 72 [59, 82] | 72 [56, 80] | 0.360 |
| Female sex, no. (%) | 400 (42.9) | 151 (50.8) | 0.019 |
| Hospital days |  |  |  |
| Total no. | 4933 | 1683 |  |
| Per hospitalization (median [IQR]) | 4 [2, 7] | 5 [3, 7] | 0.053 |
| In multi-bed rooms, no. (%) | 4563 (92%) | 1367 (81%) | < 0.001 |
| Admission* |  |  |  |
| With respiratory precautions, no. (%) | 192 (20.7) | 4 (1.3) | < 0.001 |
| With detection of respiratory virus, no. (% of all patients with respiratory precautions) | 104 (54%) | 4 (100%) | < 0.001 |
| With detection of Influenza/RSV, no. (% of all patients with respiratory precautions) | 102 (53%) | 4 (100%) | < 0.001 |

Legend: [IQR] = interquartile range; no. = number; * Admission: first two days of hospitalisation, i.e. day 0, 1 or 2

**Supplementary Table S5**

**Detailed analysis of all microbiologically proven HARVI**

| **Regular wards** | **Virus** | **Sex, age (y)** | **Time to diagnosis (days after hospitalisation)** | **Symptoms** | **Presumed mode of transmission** |
| --- | --- | --- | --- | --- | --- |
| HARVI 1 | Influenza A | female, 74y | 4 | cough | Cannot be determined, hospitalisation in multi-bed room (4 beds), no fellow patient known to have respiratory symptoms |
| HARVI 2 | Influenza A | female, 75y | 5 | cough, fever | Contact with another patient with Influenza A (resided in multi-bed room where a fellow patient had a delayed diagnosis of infection with Influenza A) |
| **DroPS wards** |  |  |  |  |  |
| HARVI 1 | Influenza A | female, 86y | 5 | cough | Cannot be determined, hospitalisation in multi-bed room (2 beds), no fellow patient known to have respiratory symptoms |
| HARVI 2 | RSV | female, 86y | 11 | cough | Fellow patient (index) with RSV did not fully comply with DroPS measures: did communicate closely at bedsite of patient with HARVI 2, according to later reports of the patient with HARVI 2. |
| HARVI 3 | Human Coronavirus | male, 84y | 12 | cough | Cannot be determined, hospitalisation in multi-bed room (2 beds), no fellow patient known to have respiratory symptoms |
| HARVI 4 | Human Coronavirus | female, 47y | 18 | (cough) | Cannot be determined, hospitalisation in multi-bed room (2 beds), no fellow patient known to have respiratory symptoms |
| HARVI 5 | Human Coronavirus, Rhinovirus | male, 77y | 6 | cough | Cannot be determined, hospitalisation in multi-bed room (4 beds), no fellow patient known to have respiratory symptoms |
